# Plasmonic hot electron transport drives nano-localized chemistry

**DOI:** 10.1038/ncomms14880

**Published:** 2017-03-28

**Authors:** Emiliano Cortés, Wei Xie, Javier Cambiasso, Adam S. Jermyn, Ravishankar Sundararaman, Prineha Narang, Sebastian Schlücker, Stefan A. Maier

**Affiliations:** 1 The Blackett Laboratory, Department of Physics, Imperial College London, London SW7 2AZ, UK; 2 Physical Chemistry I, Department of Chemistry and Center for Nanointegration Duisburg-Essen (CENIDE), University of Duisburg-Essen, Universitätsstrasse 5, 45141 Essen, Germany; 3 Key Laboratory of Advanced Energy Materials Chemistry (Ministry of Education), College of Chemistry, Nankai University, Tianjin 300071, China; 4 Institute of Astronomy, Cambridge University, Cambridge CB3 0HA, UK; 5 Faculty of Arts and Sciences, Harvard University, Cambridge, Massachusetts 02138, USA; 6 Department of Materials Science and Engineering, Rensselaer Polytechnic Institute, 110 8th street, Troy, New York 12180, USA

## Abstract

Nanoscale localization of electromagnetic fields near metallic nanostructures underpins the fundamentals and applications of plasmonics. The unavoidable energy loss from plasmon decay, initially seen as a detriment, has now expanded the scope of plasmonic applications to exploit the generated hot carriers. However, quantitative understanding of the spatial localization of these hot carriers, akin to electromagnetic near-field maps, has been elusive. Here we spatially map hot-electron-driven reduction chemistry with 15 nm resolution as a function of time and electromagnetic field polarization for different plasmonic nanostructures. We combine experiments employing a six-electron photo-recycling process that modify the terminal group of a self-assembled monolayer on plasmonic silver nanoantennas, with theoretical predictions from first-principles calculations of non-equilibrium hot-carrier transport in these systems. The resulting localization of reactive regions, determined by hot-carrier transport from high-field regions, paves the way for improving efficiency in hot-carrier extraction science and nanoscale regio-selective surface chemistry.

## Abstract (web summary)

Quantitative understanding of the spatial localization of hot carriers has been elusive. Here Cortes *et al*. spatially map hot-electron-driven reduction chemistry with 15 nm resolution as a function of time and electromagnetic field polarization for different plasmonic nanostructures.

Nanostructured materials that present plasmonic resonances enable intense light focusing, mediating electromagnetic (EM) energy transfer from the far to the near field or vice versa. Thus, these nanostructures can be considered as optical nanoantennas and are key elements in the conversion of free-space light to evanescently confined modes in nanometre-scale volumes below the diffraction limit^1^,^2^. For metals such as Au and Ag, localized surface plasmon resonances (LSPRs) in nanoantennas fall into the optical regime. Owing to the sub-wavelength character of these modes, the electric energy density is significantly higher than the magnetic counterpart. Self-sustaining EM oscillations then require an additional energy term, found in the form of a kinetic energy density of the free carriers of the metal^3^,^4^. Sub-diffraction electric field concentration at visible wavelengths in metals is only possible due to the existence of these energetic carriers, highlighting the mixed light–matter mode nature of LSPRs.

Light incident on metallic nanoantennas excites surface plasmons (SPs), which may decay to hot carriers through several mechanisms including direct interband transitions, phonon-assisted intraband transitions and geometry-assisted transitions (collectively referred to as Landau damping)^3^,^5^,^6^,^7^. In recent years, increased attention has been paid to these loss mechanisms, leading to the extension of the concept of plasmonic nanoantennas not only as sub-diffraction light-focusing objects, but also as reactive elements in the interplay between light and nanoscale materials. The possibility to extract and use hot carriers generated after the non-radiative decay of SPs has triggered new research within the field. The unavoidable drawback of losses at visible wavelengths in metals has now turned into an exciting opportunity for energy conversion, photodetection, photochemistry and photocatalysis^8^,^9^,^10^,^11^.

In spite of being very energetic, these hot carriers cannot travel over distances larger than tens of nanometres before they lose their energy via scattering^12^. After being generated, the excitations will decay on timescales ranging from a few tens of femtoseconds to picoseconds via a series of ultrafast processes such as electron–electron scattering and equilibration with the lattice by electron–phonon scattering^5^,^13^,^14^. These scattering events thermalize the carriers and bring their energies closer to the Fermi level of the metal, on average. Plasmonic hot carrier applications, on the other hand, require carriers far from the Fermi level to more efficiently drive reactions in molecules and to overcome activation barriers. Therefore, to describe hot carriers that impinge on the surface of a nanostructure for collection requires a full description of the hot carrier transport, including the spatial and temporal evolution of energy and momentum^6^,^10^,^15^. Typically, transport of excited carriers is described using one of two regimes: (i) a ballistic regime where carriers rarely scatter within the structure and (ii) a diffusive regime where several scattering events can occur within the characteristic structure dimensions. Hot carrier transport falls between the ballistic and diffusive regimes—an intermediate regime where the plasmonic structure is neither much smaller nor significantly larger than a mean free-path between sequential scattering events. Far-from-equilibrium hot carriers are a particular challenge for current Monte Carlo or Boltzmann transport methods^16^.

The carriers generated by plasmon decay impinge upon the surface of a plasmonic nanostructure to be collected, either directly or after scattering against other carriers and phonons in the metal. Collection (extraction) of hot carriers is currently accomplished via two primary methods: Schottky barrier-based devices or molecular species able to capture the electrons and/or holes from the interface. The former needs a semiconductor at the interface of the metallic nanostructure or antenna capable of accepting the carriers into its conduction band; this method has led to important developments in applications such as photodetection^17^,^18^, nanoimaging^19^ and artificial photosynthesis^20^, among others. The latter instead involves injection of hot carriers from the metal structure into available orbitals of nearby adsorbates^21^. Essentially, the proposed mechanism for plasmon-driven chemistry involves the injection of an electron from the metal into an anti-bonding state of an adsorbed molecule, causing either desorption or the dissociation of a bond in the adsorbate^9^,^22^. Depending on the potential energy landscape of these transient ions they can further react on the metal surface^23^ or diffuse and react later on in solution^24^. Hot-electron-mediated reactions at Al, Ag and Au plasmonic interfaces have been achieved by using these ideas, enabling such encouraging work as O_2_ and H_2_ dissociation, among others^25^,^26^,^27^.

Despite this significant progress in our understanding of hot-carrier generation, relaxation dynamics and extraction, there are still many open questions that need to be addressed. Among them, the possibility of mapping the reactivity of plasmonic antennas with nanometre resolution is critical, as it would guide the efficient design and fabrication of reactive nanoantennas for plasmon-induced energy conversion or photocatalysis, for example ref. 28. Although theoretical and experimental capabilities developed in nanophotonics to accurately predict and measure the electric near-field distribution in plasmonic antennas have enabled countless applications that accelerated the field of plasmonics to its current mature state^1^,^29^, a thorough understanding in the context of interfacial hot-carrier distributions is still lacking. The local reactivity of these systems is dictated by a combination of hot-carrier generation, distribution and extraction, and therefore multiple processes need to be taken into account in the experimental design and theoretical modelling.

In this report we present experimental and theoretical results of hot-electron-driven reactivity mapping with nanometre resolution. We use the ability of hot electrons to locally reduce the terminal group of a self-assembled molecular layer that covers the surface of a nanoantenna. This modification is spatially localized using 15 nm nanoparticles (NPs) specially designed to react with the converted molecules only. Scanning electron microscopic (SEM) imaging is used to record the position of the reporter NPs after different time periods of light illumination and hence plasmon excitation, thus progressively mapping the reactivity of the nanoantennas. Geometry-dependent differences have been observed between Ag bow ties (BTs) and rounded Ag bar-dimers (BDs) antennas. We give physical insight into the experimentally observed spatial reactivity distributions with the aid of an efficient computational technique that we developed to predict hot carrier transport with spatial and energy resolution in complex plasmonic nanostructures from first principles. Our results are essential to understand the efficiency of plasmonic energy conversion and plasmonic hot carrier extraction using molecular species. The transport method developed here for non-equilibrium carriers is an important tool in the prediction and explanation of this complex dynamical process. Using this method, we present quantitative theoretical predictions for hot carrier transport in Ag BTs that are in agreement with the experimental results. The combination of top-down and bottom-up fabrication approaches, selective surface chemistry and theoretical calculations of the transport of non-equilibrium carriers has allowed us to show that nanoscale regions with high EM fields in high-curvature areas are the most reactive locations within plasmonic antennas. Hot-electron-driven nanoscale patterning of surface chemistry in plasmonic antennas can now be employed for self-guided positioning of catalytic materials or molecules to highly reactive regions (reactive spots) and for the selective modification of high EM field regions (hot spots).

## Results

### Hot-electron-mediated local surface chemistry modification

**Figure 1:**
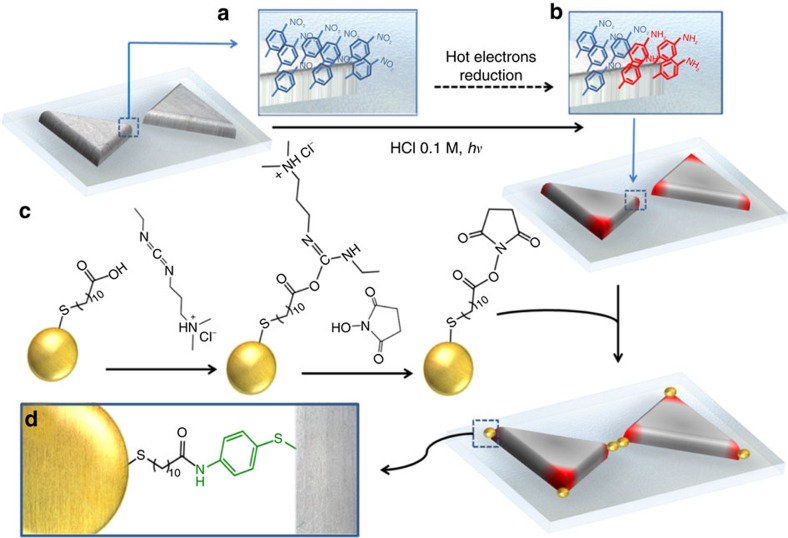
Scheme of the local surface chemistry modification and AuNPs tracking approach. (**a**) Ag nanoantennas were modified overnight with 1 mM ethanolic solution of 4-NTP. Several ethanol/water washing steps were performed on each sample. (**b**) 4-NTP-coated antennas were immersed in 0.1 M HCl solution and illuminated for different times at their LSPR wavelength (633 nm) with a power density of 1 W cm^−2^. Samples were rinsed with water and immediately dipped in the activated AuNP suspension. (**c**) AuNPs (15 nm) coated with 11-mercaptoundecanoic acid (MUA) as a capping layer were suspended in HEPES buffer and mixed with 1 mM EDC and 1 mM NHS, and left to react for 30 min followed by two purification centrifugation steps. (**d**) The activated and purified AuNPs were left in contact with the hot-electron-converted Ag antennas to react overnight, thus creating the amide (–NH–C=O) bond. Several washing steps with HEPES buffer and water were performed before 2 nm of Pt were sputtered for SEM imaging.

Let us begin by describing the experimental approach to trace reactivity in plasmonic antennas (Fig. 1). Briefly, Ag nanoantennas on quartz substrates were fabricated by electron-beam lithography. Their spectral features, near-field distribution and exact dimensions are described below. Once fabricated and characterized, Ag nanoantennas were modified with 4-nitrothiophenol (4-NTP). As a thiolated molecule, 4-NTP forms a densely packed monolayer on the surface of the Ag antennas (Fig. 1a). When 4-NTP-coated Ag antennas are illuminated at their plasmon resonance frequency in the presence of an acid halide media (that is HCl, HBr and HI), they undergo a six-electron-mediated reduction to form 4-aminothiophenol (4-ATP)^30^. Key elements for driving this reaction are hot electrons, protons and halide anions. Hot electrons are generated within silver and transferred to molecules adsorbed on the metal surface, as shown by wavelength and power-dependent studies (see Supplementary Figs 1 and 2). Protons serve as the hydrogen source. Halide ions are required for the photorecycling of electron-donating Ag atoms. Briefly, hot holes generated on the Ag surface—upon hot-electron extraction—are filled by halide ions (that is, Cl^−^). The insoluble silver halide present on the Ag surface (that is, AgCl) undergoes a photodissociation reaction that regenerates the Ag surface. A series of control experiments (see Supplementary Fig. 3) demonstrate that the reaction is neither thermally nor photochemically induced^30^.

By selecting proper conversion times, this reaction should locally change the chemical composition of the antennas only at highly reactive regions, whereas most of the antenna would remain covered by the original (unconverted) molecules (Fig. 1b). However, extracting spatially resolved chemical information at the nanoscale from a surface by a non-invasive method is not a straightforward task. Our system is sensitive to light; therefore, tools such as tip-enhanced Raman spectroscopy should be avoided for mapping reactivity^31^. Using surface-enhanced Raman scattering (SERS) for demonstrating the chemical conversion is also not conclusively meaningful, as only molecules in the EM hotspots can be detected due to dominance of the EM field enhancement^32^. In our approach we used 15 nm AuNPs functionalized with 11-mercaptoundecanoic acid and the well-known 1-Ethyl-3-(3-dimethylaminopropyl)-carbodiimide/N-hydroxysuccinimide (EDC/NHS) reaction (Fig. 1c) to report on the hot-electron conversion (see Methods for details). This highly specific and high-yield coupling reaction between the terminal carboxylic acid group in the AuNPs and the terminal amino (converted) groups in the Ag antennas is used to form an amide bond^33^ that serves as a reporter of the local hot-electron reactivity on the antennas (Fig. 1d). High-resolution SEM imaging is finally performed to track the position of the AuNPs in the Ag antennas. We analysed SEM images of the Au particles attached to 100 Ag antennas, illuminated for different lengths of time with different incident polarizations and geometries, to reveal the local hot-electron reactivity in these systems.

The fact that the NPs were added after illumination (under dark conditions), and that they were guided only by the local chemical reactivity of the antennas, allowed us to avoid high EM field-trapping effects that could have biased the results^34^,^35^. Indeed, tracking reactive regions in plasmonic antennas during illumination should be avoided, as high EM fields can have significant effects on the local concentration of reactants, masking hot-carrier reactive spots with EM field hotspots. Deposition of conducting reporters such as polymers or metal ions can generate an additional channel for electron migration, also biasing the results to trace the reactive spots in plasmonic antennas^36^,^37^,^38^. Recent results on plasmon-induced anisotropic growth of gold nanoprisms have shown that hot electrons cannot directly reduce metal ions due incommensurate timescales between the hot-electron lifetime and the slow kinetics of metal ions reduction. On the contrary, reduction only takes place in this system assisted by chemical species—such as polyvinylpyrrolidone—adsorbed on the AuNP surface that guide and prolong the hot electron lifetime and foster the reduction reaction^38^. Diffusion limitations of the reactive species to the surface of the antenna should be also considered due to the very short lifetime of the hot carriers. All these issues need to be taken into account, to properly address the problem of tracing the reactive spots in plasmonic antennas. In our system we have a homogeneous self-assembled monolayer covering the entire surface of the antennas. Thus, the whole surface is equally reactive. Furthermore, this experimental approach avoids diffusion problems of the reactive species utilized to trace the reactive spots.

**Figure 2:**
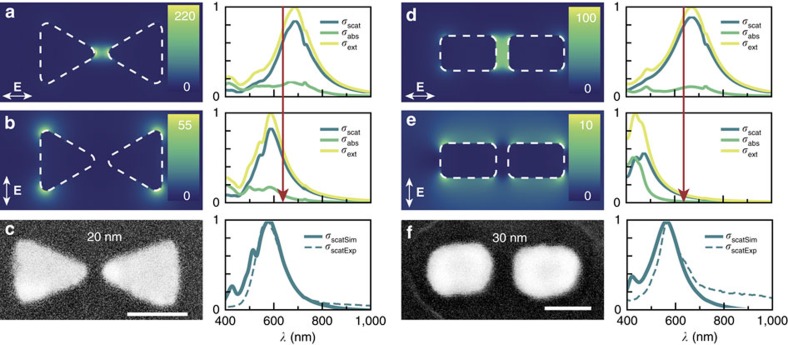
Plasmonic response of Ag BTs and Ag BD antennas. (**a**,**b**) FDTD simulations of the near-field distribution for a Ag BT antenna at 633 nm in water for parallel (**a**) and perpendicular (**b**) polarized illumination. Colour scale bars represent the field enhancement (|**E**|/|**E**_0_|)^2^ values obtained in each case. Simulated scattering (blue), absorption (green) and extinction (yellow) spectra for these polarizations are shown to the right. Red arrow highlights the laser wavelength used in the conversion experiments (633 nm). (**c**) SEM image of a Ag BT antenna (gap 20 nm), and simulated (full line) and measured (dotted line) single-antenna scattering spectra in air. Scale bar, 80 nm. (**d**,**e**) FDTD simulations of the near-field distribution for a Ag BD antenna at 633 nm in water for parallel (**d**) and perpendicular (**e**) polarized illumination. Colour scale bars represent the field enhancement (|**E**|/|**E**_0_|)^2^ values obtained in each case. Simulated scattering (blue), absorption (green) and extinction (yellow) spectra. Red arrow highlights the laser wavelength used in the conversion experiments (633 nm). (**f**) SEM image of a Ag BD antenna (gap 30 nm), and simulated (full line) and measured (dotted line) single-antenna scattering spectra in air. Scale bar, 100 nm.

We fabricated two different types of antennas: Ag BT and Ag BD. Finite-difference time-domain (FDTD) simulations were conducted to determine the range of Ag antenna sizes that exhibit a plasmon resonance at 633 nm in water (see Methods for simulation details). Ag antennas were then fabricated by electron-beam lithography (see Methods for details on fabrication and characterization). Each sample is composed of an array of 100 antennas of the same dimensions. Ag markers (1 μm in size) were added at each corner of the array and served as non-plasmonic control surfaces. Figure 2 shows the FDTD simulated near-field electric field distribution for the obtained dimensions of Ag BT (Ag BD) antennas illuminated at 633 nm in water for parallel (Fig. 2a,d) and perpendicular (Fig. 2b,e) polarizations. Colour scale bars represent the electric field enhancement (|**E**|/|**E**_0_|)^2^ values obtained in each case. We also show the simulated scattering, absorption and extinction spectra for each case. Figure 2c,f shows typical SEM images for the obtained Ag BT (Ag BD) antennas and also single-antenna scattering spectra measured in air for each case. In this way, we have arrays of antennas with different shapes and tuned spectral characteristics to probe the localization-conversion experiments. We have measured single-antenna scattering spectra over a number of antennas on each array, confirming a highly uniform spectral response (see Supplementary Fig. 4).

### Experimental mapping of hot-electron conversion

Once the antennas were fabricated and characterized, we performed the conversion experiments as described in Fig. 1. However, a series of control experiments were necessary, first to show that conversion takes place at the single antenna level, and also that the proposed surface chemistry reactions were efficient and specific enough to trace the hot-electron converted molecules.

**Figure 3:**
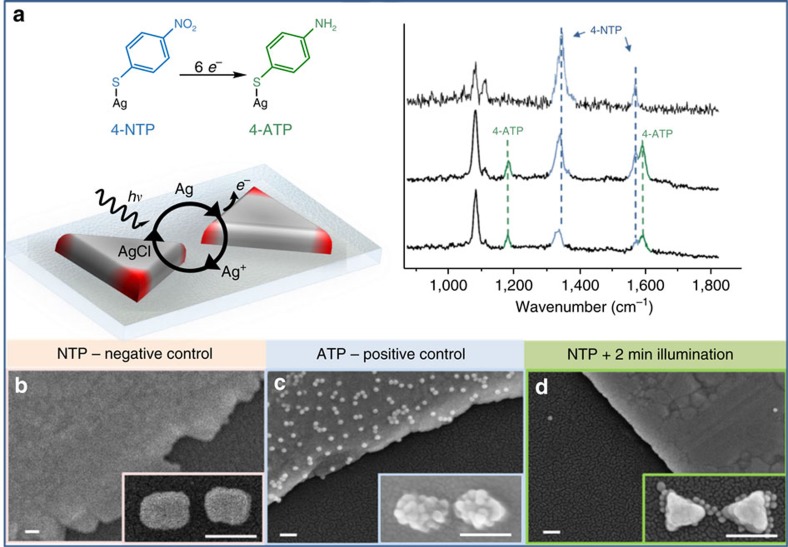
*In situ* label-free SERS monitoring of hot-electron-mediated reduction and control experiments. (**a**) Single-antenna SERS detection of hot-electron reduction from 4-NTP to 4-ATP in the presence of 0.1 M HCl, *λ*=633 nm, power 1 mW, integration time 5 s. Time-dependent spectra highlighting the conversion from 4-NTP (top spectrum) to 4-ATP (bottom spectra). (**b**) SEM images of the negative control experiment: sample was coated with 4-NTP and left to react with the activated 15 nm AuNPs. No particles were detected on either the Ag film or on the Ag antennas (inset). (**c**) SEM images of the positive control experiment: sample was coated with 4-ATP and left to react with the activated 15 nm AuNPs. After several washes of the sample with HEPES buffer and water, the amide reaction was detected both on the Ag film and the Ag antennas (inset). (**d**) SEM images of the partial-conversion experiment: Ag antennas were coated with 4-NTP and illuminated at 633 nm for 2 min in 0.1 M HCl at a power density of 1 mW cm^−2^. AuNPs were found attached only to the Ag antennas, whereas no particles were found on the substrate or in the Ag film. All the samples were treated under exactly the same conditions, followed by the same washing steps before 2–3 nm Pt coating for SEM imaging. Scale bars, 100 nm for all images.

To show that conversion takes place under our experimental conditions, we performed single-antenna SERS to detect the conversion from 4-NTP to 4-ATP (Fig. 3a). High EM field confinement in our antennas (Fig. 2) allowed label-free monitoring of the reaction by *in situ* SERS spectroscopy. By employing 4-NTP-coated Ag antennas in the presence of 0.1 M HCl and by using a 10 μW diffraction-limited spot at *λ*=633 nm (that is, at the plasmon resonance of our antennas), the C–C and C–H stretching bands of 4-ATP appeared at ∼1,590 and ∼1,180 cm^−1^, respectively (Fig. 3a). Temporal spectral series showed that 4-NTP Raman peaks decreased, whereas 4-ATP peaks increased. Raman cross-sections (*σ*) for both molecules are not the same (*σ*_4-NTP_>*σ*_4-ATP_); thus, it is not possible to make a linear correlation of this behaviour. However, they followed the expected trend—shifting the laser excitation wavelength away from *λ*_max_ of the plasmon peak leads to lower reduction activity (see Supplementary Fig. 2). These results confirm that the six-hot-electron reduction reaction can proceed in our system to completely convert 4-NTP into 4-ATP.

After confirming the conversion reaction in our antennas, we moved towards testing the surface chemistry reactions. A negative control experiment was performed by incubating a 4-NTP-modified Ag antenna sample with the activated 15 nm AuNPs (activation proceeds as described in Fig. 1 by EDC/NHS chemistry). SEM imaging of the antennas and surrounding Ag films (markers) showed that, as expected, no reporter particles remain attached on either the Ag antennas, the Ag film or the quartz surface (Fig. 3b). The same result was obtained using a bare Ag film as a further negative control (see Supplementary Fig. 5). By contrast, when the same experiment was performed under exactly the same conditions on a 4-ATP-coated sample, we detected 15 nm AuNPs attached to both the Ag film (see Supplementary Fig. 5 for larger areas) and the Ag antennas with no trace of the particles on the quartz substrate (Fig. 3c). These two control experiments indicate that the reaction is highly specific to the presence of 4-ATP, and that the amide-bond formation proceeds at a very high yield under these experimental conditions (inset Fig. 3c). Other less effective reactions and conditions were also tested, as well as attempts to reduce the size of the AuNPs even further (see Supplementary Note 1). Finally, we performed a control experiment on a partially converted sample: a 4-NTP-functionalized Ag-antenna array was illuminated at 633 nm with a 0.5 mm spot at 1 W for 2 min in 0.1 M HCl and 15 nm activated AuNPs were added right after finishing the illumination. The markers and the entire array were therefore illuminated at the same time. As shown in Fig. 3d, some NPs appeared to attach only to the antenna (that is, no particles on the Ag film or on the quartz substrate). This last control shows that LSPRs are necessary for the reaction to take place^39^. The probability of interband optical absorption is very low for Ag at this excitation wavelength (633 nm) and therefore chemical conversion mediated by this absorption method should not occur on the Ag film^40^; only carriers derived from the non-radiative decay of LSPRs should contribute to the reduction of 4-NTP. These experiments demonstrate that the method described in Fig. 1 is suitable to monitor the local changes in reactivity of the plasmonic antennas by hot-electron-mediated reduction chemistry.

### Hot carrier transport and spatial distributions

To obtain physically meaningful results, the experimental methodology described above relies on a highly site-specific extraction of energetic carriers from the nanoantennas. We investigate this assumption now theoretically from first principles. It is noteworthy that decay of SPs generates hot carriers through several mechanisms including direct interband transitions, phonon-assisted intraband transitions and geometry-assisted intraband transitions, which we have presented in previous work^7^,^13^.

The spatial dependence of hot-carrier driven reduction reactions depends on the spatial distribution of sufficiently energetic hot electrons reaching the surface. We emphasize that this distribution depends both on the initial spatial and energy distributions of hot carriers generated by plasmon decay and the subsequent transport of the generated electrons to the surface.

Our theoretical predictions start with the initial spatial and energy distribution of hot carriers generated by plasmon decay,


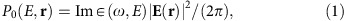


where Im∈(ω,*E*) is the *ab initio* frequency-dependent imaginary dielectric function, resolved by the carrier energy *E* generated by the responsible electronic transition and **E**(**r**) is the electric field distribution inside the metal upon illumination obtained from EM simulations. The calculated dielectric function includes contributions due to direct interband transitions^7^ and phonon-assisted intraband transitions^13^ in general, although only the latter contribute to carrier generation in silver at 633 nm illumination (≈2 eV photons, below the interband threshold ≈3.6 eV).

We then calculate the carrier flux *φ*_*n*_(*E*,**r**) reaching the surface after *n* scattering events as


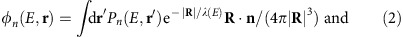



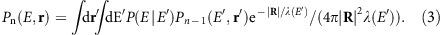


Above, *P*_n_(*E*,**r**) is the distribution of hot carriers generated after *n* scattering events, **R**:=**r**−**r**′ is the separation between source and target points and **n** is the unit surface normal vector. We use *ab initio* calculations of electron–electron and electron–phonon scattering to determine the energy-dependent carrier mean-free path *λ*(*E*) and the probability *P*(*E*|*E*′) that the scattering of a carrier of energy *E*′ generates a carrier of energy *E*. We assume that the number of generated hot carriers is negligible compared with the number of thermal electrons in the metal, such that these quantities depend only on the carrier energy and the background distribution. The final hot carrier flux *φ*(*E*,**r**)=*∑*_*n*_ *φ*_*n*_(*E,***r**) accounts for carriers that reach the surface without scattering and after an arbitrary number of scattering events. This approach efficiently solves a linearized Boltzmann equation with an *ab initio* collision integral^5^, assuming that each scattering event randomizes the carrier momentum, which is an excellent approximation for carriers with |*E*−*E*_f_|<<*E*_f_, the Fermi energy (Jermyn *et al*., manuscript in preparation). See Methods for further details.

**Figure 4:**
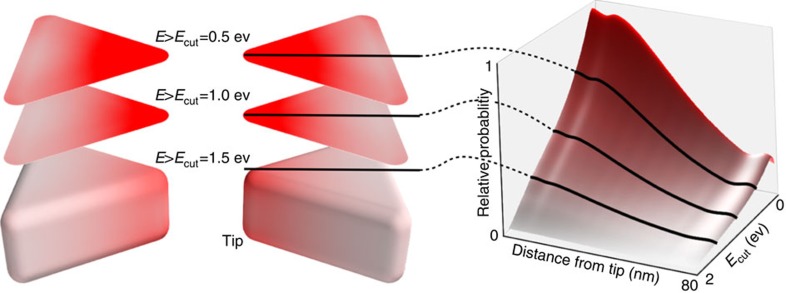
Theoretical predictions for spatially resolved energy distributions of plasmonic hot carriers. These predictions are based on *ab initio* calculations of hot carrier generation including phonon-assisted intraband excitations and a transport model that accounts for multiple scattering events and the energy-dependent electron mean-free path from *ab initio* calulations. The left panel shows the relative flux of hot electrons with energy greater than a threshold *E*_cut_ (relative to the Fermi level of silver) on the surface of a Ag BT antenna illuminated at resonance (633 nm). The right panel shows the corresponding distribution as a function of *E*_cut_, averaged over planes of constant distance from the tip. The probability drops linearly with increasing *E*_cut_ and exponentially with distance from the tip due to the plasmon field distribution inside the metal and the transport of hot carriers from the point of generation to the surface. Higher *E*_cut_ results in lower hot carrier flux, but greater spatial resolution.

Figure 4 shows the predicted hot carrier flux reaching the surface of an Ag BT antenna. We show the spatially resolved probabilities of carriers with energy *E* greater than a threshold *E*_cut_, which can for example be interpreted as the minimum carrier energy required to drive a chemical reaction. The highest hot carrier flux is at the tip of the BT antenna where the field is strongest and then decays exponentially away from the tip. The exponential decay length depends both on the plasmon field distribution (as we have shown previously) inside the metal (the skin-depth length scale) and the mean-free path of the hot carriers. Hot electrons of lower energy reach further from the field hotspots due to their longer mean-free path and because they can be generated after multiple scattering events, whereas higher energy electrons remain localized closer to the field hotspots. Thermalization of high energy carriers increases the probability for charge transfer events to molecular states, this being a resonant process. As such, the high *E*_cut_ necessary to generate a sufficient density of extracted carriers to drive the six-electron reduction reaction results in the high spatial resolution of the chemistry observed in the experiments as follows.

**Figure 5:**
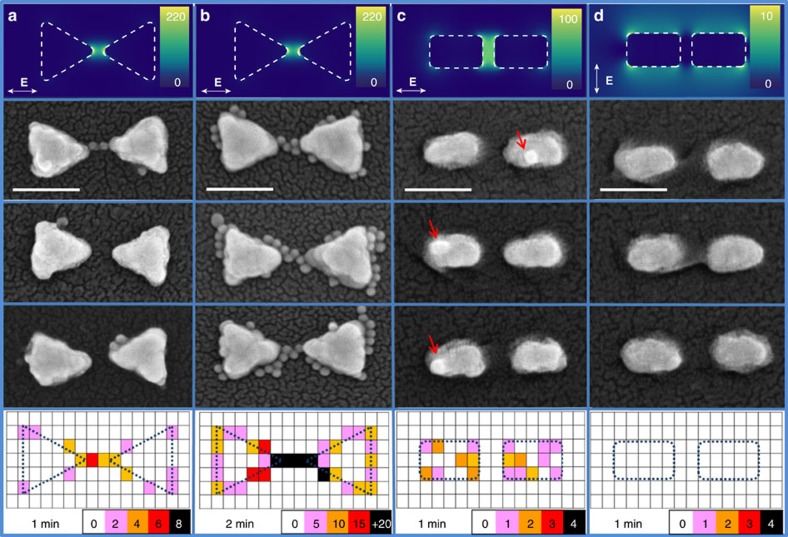
Mapping hot-electron conversion in Ag antennas for different illumination times and polarizations. Au reporter particle binding to 4-NTP-coated Ag BTs after (**a**) 1 min and (**b**) 2 min of illumination with parallel polarization at 633 nm in 0.1 M HCl. Au reporter particle binding to 4-NTP-coated Ag BDs conversion after 1 min of (**c**) parallel polarized illumination and (**d**) perpendicular polarized illumination at 633 nm in 0.1 M HCl. (**a**–**d**) Top panels show the near-field distribution calculated via FDTD simulations for each case at 633 nm. Middle panels illustrate representative SEM images of the localized 15 nm AuNPs forming an amide bond with the converted molecules on the antenna (4-ATP). Scale bars, 100 nm. Bottom panels show the histograms (collapsed localizations maps) over 100 antennas, for each experimental condition. The position of the particles and the antennas were recorded by SEM inspection. Only antennas orientated between 0° and 20° respect to the incident polarization were taken into account (see Supplementary Fig. 8). Colour bar indicates the frequency of appearance of AuNPs localized in each pixel after performing the statistical analysis (that is, summed over all the antennas under the same experimental conditions). For example, in bottom of **b**, 0 (white) means 0 AuNP localized in those pixels; 5 (violet) means pixels with 1 to 5 AuNPs; 10 (orange) means pixels with 6 to 10 AuNPs; 15 (red) means pixels with 11 to 15 AuNPs and +20 (black) means pixels with 16 to over 20 AuNPs. The size of the mesh (15 nm) was chosen so as to match with the size of the reporters (AuNPs).

Figure 5 presents the results of our efforts to localize hot-electron-mediated reactions. We evaluated geometry, time and polarization dependence on hot-electron transfer to 4-NTP in Ag antennas. Figure 5a shows the results for parallel illumination (*λ*=633 nm) in Ag BT after 1 min of conversion (in 0.1 M HCl). Representative SEM images clearly point towards the fact that the hotspot tips of the structure are the most reactive regions (that is, they exhibit preferential binding of the Au reporter NPs). A histogram containing the localization of reporter NPs from all 100 antennas in the array is presented at the bottom of the column (see Supplementary Note 2 and Supplementary Figs 6–8 for further details). We repeat that the 15 nm AuNP reporters are added after the conversion (with no illumination); thus, they are only guided by the surface chemistry reactivity. This fact allows us to avoid optical trapping and high EM field effects that could potentially mask the results. Our observations are in line with previous and current theoretical considerations on carrier extraction from metallic nanoantennas^6^. Photoemission spectroscopy of essentially free electrons from plasmonic antennas^41^ has shown preferential emission at field hotspot regions; here we demonstrate similar site-selective extraction of bound carriers to locally drive surface chemistry, although we emphasize we are not collecting photo-emitted electrons. Once carriers have been generated, the possibility that they reach the surface and escape (that is, transfer to the adsorbed 4-NTP molecule) before the onset of significant energy loss occurs inside the material is strongly dependent on the curvature of the structure. This explains the strong reactivity observed near the tips, consistent with our calculations presented in Fig. 4. These results are in line with the observation of Moskovits *et al*.^36^, as the randomization of the momenta of hot electrons occurs on a shorter time scale, with the first scattering event, than the time for complete electron thermalization; therefore, the geometry of the antenna becomes the crucial parameter for hot carriers to reach the surface. Nanoscale confinement of carriers also plays an important role for their final extraction capabilities, with importance of subtler aspects such as defects and/or crystal orientation within the metal nanoantenna. We would expect an anisotropic distribution in the localization of the reporter-AuNPs if carrier collection was dominated by defects in our polycrystalline structures. However, in our experiments, large sample size statistics show clear geometry-dependent reactive spots for short illumination times (Fig. 5a bottom panel). Hot electron travel distances to their final extraction points are greatly reduced in our nanoantennas compared to recent results shown for a 3 μm semiconductor wire, where defects and impurities dominate the extraction of carriers exited over the band gap^28^. For this illumination time, where we have few particles per antenna, we have noticed a strong dependence in the localization map (histogram) regarding the orientation of the antenna with respect to the illumination polarization (see Supplementary Fig. 8). Antennas presenting tilts over 20° (respect to the main axes) present also AuNPs reporters at the corners of the antenna, see the second SEM image in Fig. 5a. These results are in line with our theoretical predictions and polarization-dependent field distribution (Fig. 2a,b and Supplementary Fig. 8). We also noticed a strong time dependence in the evolution of the conversion reaction. Shorter illumination times (that is, 30 s) resulted in no particles localized. These conclusions are further supported by the following additional observations.

Figure 5b shows the localized particles in Ag BTs after 2 min of illumination with parallel polarization. The progression of the reaction can be followed and we note that after the central tips, the edges and corners seem to be the most reactive regions within this geometry. For a carrier generated inside the material, the probability of reaching the surface is higher at the edges/corners in comparison with the flat-topped surface due to curvature. For Ag BTs, our results imply then that reactivity is highest at sharp tips and lowest on flat planar sections of the structure. In particular, this requires feature sizes that are smaller than the hot carrier mean-free path, an energy-dependent quantity that results in ≈10 nm for electrons 2 eV above the Fermi level in silver^13^. This indicates that in our six-electron reaction the spatial confinement is governed by a combination of the density of carriers that reach the surface in a given time period and the energy cutoff. As shown, for longer illumination times we can detect particles far from where they are primarily generated from which we infer that very low-energy carriers are able to convert 4-NTP to 4-ATP. Our calculations point towards that spatial confinement should also be achievable with reactions involving higher energetic barriers. Then, the number of electrons involved or the energy barrier of the reaction should act as spatial filters for hot-carrier-driven process. However, the site-specific spatial confinement can also be lost if the final acceptor of the electrons (coating the antennas) delocalize them, as recently shown for polyvinylpyrrolidone-induced anisotropic growth of Au nanoprisms in plasmon-driven synthesis^38^.

To evaluate the geometry-dependent reactivity in Fig. 5c, we show representative SEM images for Ag BDs after 1 min of parallel illumination. For these rounded structures without sharp edges or corners, the localization maps (Fig. 5c, bottom panel) differ dramatically from those observed for Ag BTs. In this case, the reporter particles are always found on top of the antennas, with no preference for the surrounding regions. Based on these results, if we imagine two nanostructures with the same absorption cross-section (*σ*_Abs_) at a given resonant wavelength (*λ*) but shaped in different geometries, the one with sharper features would produce hot carriers that should be more efficiently extracted before their energy is lost. This example shows that AgCl dissociation (recycling mechanism) occurs with the same probability all over the surface once the initial electron–hole pairs have been generated. Finally, with perpendicularly polarized illumination, we could not detect any attached particles over the entire array (Fig. 5d). This is in line with our findings with the Ag film control experiment (Fig. 3d), demonstrating that non-radiative plasmon decay is the main source of hot electrons driving the reaction. As expected, absorption in these structures for this polarization is greatly reduced (Fig. 2e), suggesting that the strength of the EM field is related to the yield of the reaction^39^. This experiment can be rationalized as a power-dependent one^25^.

Our results clearly establish that site-selective extraction of hot carriers is the main consideration in determining the reactivity of the system, not hot carrier generation alone. Although hot-carrier induced dissociation reactions, involving diatomic species such as H_2_, O_2_ and CO had been recently described at the molecular level^23^,^25^,^26^,^42^, we note that our reaction undergoes a six hot electron and a six protons (H^+^) transfer to generate the final desired product (4-ATP) and two water molecules (H_2_O) as side products^30^. A complex molecular mechanistic study is out of the scope of the presented work^43^. However, density functional theory calculations for the first intermediate species show that proton transfer should be the limiting step with an estimated energy of Δ*G*≈0.35 eV, compared with electron transfer that has essentially no activation barrier (see Supplementary Fig. 9). We do require the transfer of six individual electrons, that is, a single electron transfer event is not sufficient to report on the conversion. However, the applicability of our results is then broadened as the reaction can proceed with electrons of any energy, but only where they are generated in high enough quantities, as already described. The results should therefore more accurately reflect the locations where carriers are preferentially reaching the surface, thus highlighting locations of high reactivity in these nanoantennas (in a molecule or energy-independent manner). We note that the ability to locally tune the surface chemistry and reactivity of nanoantennas could open important avenues for reactive-spot and hotspot modification.

## Discussion

Low efficiency in hot-carrier induced chemical reactions is the main unsolved problem that can pave the way in the field to large scale application of these concepts^20^,^44^. Figures 4 and 5 show a strong spatial-energy dependence of the generated carriers and their extraction, both from first-principle calculations and experiments. Reactions requiring highly energetic carriers will proceed only in a very small fraction of the antenna's surface. In addition, reactions involving high density of electrons will be strongly localized. Thus, these results can open new avenues for the design of much more efficient nanoscale plasmonic systems for hot-carrier transport-driven chemical reactions^28^,^45^. Our results on the transport and nanoscale localization of extracted carriers show that by tuning the strength of the EM field within the plasmonic antenna and by reducing the mean free path of the carriers with high-local curvature tips, we expect dramatic enhancement in the efficiency of hot-carrier-induced chemical reactions and/or Schottky barrier photodetection and energy conversion approaches.

As noted previously, the method used here to map the reactivity of plasmonic antennas relies on the ability to locally change the surface chemical properties of the antennas by selective hot-electron mediated reduction of the molecular layer (Fig. 1). The ability to have highly localized differential chemical reactivity on the nanoantenna's surface can be further used to guide molecules, proteins and other nanomaterials (that is, catalytic NPs) to specific regions of the nanoantenna^46^. For instance, small catalytic particles, which by themselves do not have a significant far-field cross-section, can be incorporated to larger size structures that possess higher electric dipole moments to perform as nanoantennas^47^,^48^.

Furthermore, through careful design of antennas with sharp tips present only at the structure's EM hotspots, this method could serve as an efficient, self-guided way of modifying only high EM field regions, while keeping most of the antenna chemically passive (that is, not reactive). In this way we can access both the most reactive spots (in terms of hot electron transfer) and the most plasmonically active regions (hotspots) of the nanoantennas. Attaching molecules to regions with the highest hot electrons extraction and/or the highest EM fields can further improve the efficiency of such photon or electron driven processes as photocatalysis, (bio)sensing, energy conversion and imaging. This easy, fast and cheap strategy could serve as a selective and large-scale method of positioning molecules/nanomaterials in a variety of plasmonic nanoantenna reactive spots or hot spots. Photo-polymerization^49^, three-photon absorption of disulphuric species^50^ or local solvent heating strategy^51^, among others, have been recently employed for high EM field hotspots modification. Here we have demonstrated that reactive spots can also be selectively accessed (Supplementary Fig. 10). We have shown this proof of concept by using 4-NTP-coated Ag antennas and 15 nm AuNPs capped with a carboxylic-acid terminated molecular layer, although, in principle, any amino reactive reaction may be used. By completing the reaction with a reducing agent such as sodium borohydride (NaBH_4_), this method should also be useful on Au nanoantennas, where selective hot-electrons reduction from 4-NTP to 4,4′-dimercaptoazobenzene takes place^52^. Electrostatic-guided interaction can also be exploited due inherent differences between amino and nitro terminal groups.

We have shown the ability to map a hot-electron reduction reaction on Ag nanoantennas with 15 nm spatial resolution and corroborated the spatially highly confined surface chemistry by first-principles calculations of hot carrier generation and transport. Our results progressively traced the reactivity in plasmonic antennas, highlighting strong dependence of the reactivity on the EM field distribution within the metal. Our theoretical treatment of plasmonic hot carrier generation and transport confirm nanoscale localization of high-density carrier regions required to drive this multi-electron chemical reaction and predict an inverse relation between collected carrier density and transport distance. Polarization-resolved experiments demonstrate that the high EM field intensities and the absorption of the nanoantennas are necessary to drive the reaction. Improved design of highly reactive and efficient antennas should benefit from these results. As we have shown, local surface chemistry can be tuned by employing this method, opening new possibilities for accessing regions of highly concentrated photon and electron densities. Positioning of nanomaterials or molecules in these regions is now possible and should boost research and applications in plasmonic hot-carrier science.

## Methods

### Ag antennas modification and chemical activation of AuNPs

Samples were immersed overnight in 1 mM ethanolic solution of the corresponding thiols: 4-NTP or 4-ATP. Thiols were purchased from Sigma-Aldrich and used as received. Several ethanol/water washing steps were performed on the sample after 4-NTP coating. AuNPs (15 nm) were purchased from BBI Solution and modified overnight in water with 11-mercaptoundecanoic acid. Three centrifugation steps at 10,000 r.p.m. for 20 min were completed, to purify the particles. Re-suspension was carried out in HEPES buffer. After that, a HEPES solution of 0.1 mM EDC and 0.25 mM NHS were added to the NPs and left to react for 20 min. One centrifugation step at 10,000 r.p.m. for 20 min was completed, to purify the particles. Re-suspension was carried out in HEPES buffer.

### Coupling reaction between AuNPs and Ag antennas

Right after activation the 15 nm AuNPs were put in contact with the Ag antennas samples and left to react overnight at room temperature and dark environment. Three washing steps with HEPES buffer and water were performed before Pt coating for SEM imaging. We have observed that washing steps are critical for complete removal of unbounded particles. Other reactions and conditions were also tested to report on the local conversion but their specificity and/or yields were not as good as the one reported here (see Supplementary Note 1).

### FDTD calculations of the optical response of the antennas

Simulations of structures were performed using Lumerical FDTD Solutions. Different antenna lengths and gap sizes were evaluated to yield resonances in the desired wavelength range (close to 633 nm). The antennas were simulated on a SiO_2_ substrate and surrounded either by air or by water. The antenna material was Ag by McPeak *et al*.^53^ as a similar method of evaporation deposition to that described within was employed. Given the refractive properties of quartz, air, and water are relatively constant and loss-free in the studied wavelength range of 400–1,000 nm, all modelled as homogeneous wavelength-independent materials with refractive index 1.54 for quartz^54^, 1 for air^55^ and 1.324 for water^56^. A mesh step size of 1 nm was used and tests were done to assure convergence. Finally, a total-field scattered-field source was used to measure the scattering cross-section outside the source region. The plots of the scattering, absorption and extinction spectra were all normalized to the maximum value of extinction at a specific polarization configuration.

### Hot carrier generation and transport predictions

We calculate electronic states, phonons, optical and electron–phonon matrix elements, and transform them to a Wannier basis using the density functional theory software JDFTx^57^. We then calculate the carrier-energy-resolved imaginary dielectric function Imɛ(*ω*,*E*) due to direct and phonon-assisted transitions and the energy-dependent carrier mean-free path *λ*(*E*) and the resulting scattered energy distribution *P*(*E*|*E*′) due to electron–electron and electron–phonon scattering, using Fermi's golden rule^8^,^14^. We then efficiently evaluate the multi-dimensional integrals in (2) and (3) to obtain the final hot carrier flux by first evaluating the integral at specific directions of **R**=**r**−**r**′ using the 1D Green's function (exp(−*x*/*λ*)) on a tetrahedral mesh and then integrating over directions of **R** using Monte Carlo sampling with 10^4^ samples.

### Fabrication and characterization of Ag antennas

The samples were fabricated following a one-step E-Beam writing and a metal evaporation as described in the following. Quartz polished coverslips (GPE Scientific) were cleaned in three steps of 5 min each under DI Water, acetone and isopropyl alcohol (IPA). Poly(methyl methacrylate) (PMMA), 950k A4 (Microchem) was spin-coated onto the sample at 3,500 r.p.m. for 1 min, yielding a homogeneous thickness of around 200 nm. A thin layer of the conductive material Espacer 300Z (Showa Denko Europe) was spin-coated at 2,000 r.p.m. for 1 min to hinder charging under the E-beam. Following that, the samples were loaded in a Raith eLine and the structures were defined in the PMMA layer under a voltage acceleration of 20 kV, 20 μm aperture, at a working distance of around 10 mm, area step size of 6 nm and area dosage of 100 μC cm^−2^. Afterwards, the samples were developed for 30 s in a solution of 1:3 methyl isobutyl ketone: IPA, followed by a developer stopper of IPA for another 30 s. An O_2_ plasma asher was used to remove any PMMA debris in the developed holes. Then, silver was resistively evaporated in an Åmod system (Ångstrom Engineering, Inc.) at low pressure 7 × 10^−7^ Torr, fast rate of 30 Å s^−1^, resulting in a final thickness of 60 nm. The samples were left to lift-off in acetone for 24 h. Careful rinsing with acetone and IPA followed by N_2_ gun drying finalized the sample fabrication. We avoid using adhesion layers between Ag and the quartz substrate, as they can compete with the molecular layer for the extraction of the carriers^39^. SEM images were taken to ensure the final sizes matched the expected simulated sizes. The pitch of the array (for all the samples) was 2 μm (*y* direction) and 4 μm (*x* direction). After coupling the AuNPs, samples were sputtered with 2–3 nm Pt before high-quality SEM imaging. The position of the AuNPs and the antennas were recorded (by SEM), to perform the statistical analysis shown in Fig. 5. Additional statistics on the images shown in the text and the Supplementary Information can be found in the Supplementary Fig. 6. Dark-field spectra of the scattering cross-sections of the antennas in air were measured with a diffractive grating and charge-coupled device camera cooled to 70 K. The plotted spectra are corrected for the system wavelength response (by measuring the cross section of a perfect reference white rough scatterer) and also for dark and background counts. Samples were fabricated immediately before usage for hot-electron conversion experiments and 4-NTP or 4-ATP layers were formed immediately to avoid Ag oxidation.

### Data availability

All the data are available from the authors.

## Additional information

**How to cite this article:** Cortés, E. *et al*. Plasmonic hot electron transport drives nano-localized chemistry. *Nat. Commun.* **8,** 14880 doi: 10.1038/ncomms14880 (2017).

**Publisher's note:** Springer Nature remains neutral with regard to jurisdictional claims in published maps and institutional affiliations.

## Supplementary Material

Supplementary Information Supplementary Figures, Supplementary Notes and Supplementary References.

## References

[b1] Giannini V., Fernández-Domínguez A. I., Heck S. C. & Maier S. A. Plasmonic nanoantennas: fundamentals and their use in controlling the radiative properties of nanoemitters. Chem. Rev. 111, 3888–3912 (2011). 21434605 10.1021/cr1002672

[b2] Maier S. A. Plasmonics: fundamentals and Applications 1st Edn Springer US (2007).

[b3] Khurgin J. B. How to deal with the loss in plasmonics and metamaterials. Nat. Nano 10, 2–6 (2015). 10.1038/nnano.2014.310 25559961

[b4] Shalaev V. M., Douketis C., Stuckless J. T. & Moskovits M. Light-induced kinetic effects in solids. Phys. Rev. B 53, 11388–11402 (1996). 10.1103/physrevb.53.11388 9982756

[b5] Narang P., Sundararaman R. & Atwater H. A. Plasmonic hot carrier dynamics in solid-state and chemical systems for energy conversion. Nanophotonics 5, 96–111 (2016).

[b6] Manjavacas A., Liu J. G., Kulkarni V. & Nordlander P. Plasmon-induced hot carriers in metallic nanoparticles. ACS Nano 8, 7630–7638 (2014). 24960573 10.1021/nn502445f

[b7] Sundararaman R., Narang P., Jermyn A. S., Goddard Iii W. A. & Atwater H. A. Theoretical predictions for hot-carrier generation from surface plasmon decay. Nat. Commun. 5, 5788 (2014). 25511713 10.1038/ncomms6788 PMC4284641

[b8] Atwater H. A. & Polman A. Plasmonics for improved photovoltaic devices. Nat. Mater. 9, 205–213 (2010). 20168344 10.1038/nmat2629

[b9] Linic S., Aslam U., Boerigter C. & Morabito M. Photochemical transformations on plasmonic metal nanoparticles. Nat. Mater. 14, 567–576 (2015). 25990912 10.1038/nmat4281

[b10] Brongersma M. L., Halas N. J. & Nordlander P. Plasmon-induced hot carrier science and technology. Nat. Nano 10, 25–34 (2015). 10.1038/nnano.2014.311 25559968

[b11] Clavero C. Plasmon-induced hot-electron generation at nanoparticle/metal-oxide interfaces for photovoltaic and photocatalytic devices. Nat. Photonics 8, 95–103 (2014).

[b12] Scales C. & Berini P. Thin-film Schottky barrier photodetector models. IEEE J. Quant. Electron. 46, 633–643 (2010).

[b13] Brown A. M., Sundararaman R., Narang P., Goddard W. A. & Atwater H. A. Nonradiative plasmon decay and hot carrier dynamics: effects of phonons, surfaces, and geometry. ACS Nano 10, 957–966 (2016). 26654729 10.1021/acsnano.5b06199

[b14] Hartland G. V. Optical studies of dynamics in noble metal nanostructures. Chem. Rev. 111, 3858–3887 (2011). 21434614 10.1021/cr1002547

[b15] Ma J., Wang Z. & Wang L.-W. Interplay between plasmon and single-particle excitations in a metal nanocluster. Nat. Commun. 6, 10107 (2015). 26673449 10.1038/ncomms10107 PMC4703846

[b16] Savić I., Donadio D., Gygi F. & Galli G. Dimensionality and heat transport in Si-Ge superlattices. Appl. Phys. Lett. 102, 073113 (2013).

[b17] Knight M. W., Sobhani H., Nordlander P. & Halas N. J. Photodetection with active optical antennas. Science 332, 702–704 (2011). 21551059 10.1126/science.1203056

[b18] Chalabi H., Schoen D. & Brongersma M. L. Hot-electron photodetection with a plasmonic nanostripe antenna. Nano Lett. 14, 1374–1380 (2014). 24502677 10.1021/nl4044373

[b19] De Angelis F. . Nanoscale chemical mapping using three-dimensional adiabatic compression of surface plasmon polaritons. Nat. Nano 5, 67–72 (2010). 10.1038/nnano.2009.348 19935647

[b20] Mubeen S. . An autonomous photosynthetic device in which all charge carriers derive from surface plasmons. Nat. Nano 8, 247–251 (2013). 10.1038/nnano.2013.18 23435280

[b21] Linic S., Christopher P. & Ingram D. B. Plasmonic-metal nanostructures for efficient conversion of solar to chemical energy. Nat. Mater. 10, 911–921 (2011). 22109608 10.1038/nmat3151

[b22] Linic S., Christopher P., Xin H. & Marimuthu A. Catalytic and photocatalytic transformations on metal nanoparticles with targeted geometric and plasmonic properties. Acc. Chem. Res. 46, 1890–1899 (2013). 23750539 10.1021/ar3002393

[b23] Christopher P., Xin H. & Linic S. Visible-light-enhanced catalytic oxidation reactions on plasmonic silver nanostructures. Nat. Chem. 3, 467–472 (2011). 21602862 10.1038/nchem.1032

[b24] Navalon S., de Miguel M., Martin R., Alvaro M. & Garcia H. Enhancement of the catalytic activity of supported gold nanoparticles for the fenton reaction by light. J. Am. Chem. Soc. 133, 2218–2226 (2011). 21280633 10.1021/ja108816p

[b25] Christopher P., Xin H., Marimuthu A. & Linic S. Singular characteristics and unique chemical bond activation mechanisms of photocatalytic reactions on plasmonic nanostructures. Nat. Mater. 11, 1044–1050 (2012). 23178296 10.1038/nmat3454

[b26] Mukherjee S. . Hot electrons do the impossible: plasmon-induced dissociation of H_2_ on Au. Nano Lett. 13, 240–247 (2013). 23194158 10.1021/nl303940z

[b27] Zhou L. . Aluminum nanocrystals as a plasmonic photocatalyst for hydrogen dissociation. Nano Lett. 16, 1478–1484 (2016). 26799677 10.1021/acs.nanolett.5b05149

[b28] Sambur J. B. . Sub-particle reaction and photocurrent mapping to optimize catalyst-modified photoanodes. Nature 530, 77–80 (2016). 26842056 10.1038/nature16534

[b29] Halas N. J., Lal S., Chang W.-S., Link S. & Nordlander P. Plasmons in strongly coupled metallic nanostructures. Chem. Rev. 111, 3913–3961 (2011). 21542636 10.1021/cr200061k

[b30] Xie W. & Schlucker S. Hot electron-induced reduction of small molecules on photorecycling metal surfaces. Nat. Commun. 6, 7570 (2015). 26138619 10.1038/ncomms8570 PMC4506517

[b31] van Schrojenstein Lantman E. M., Deckert-Gaudig T., Mank A. J. G., Deckert V. & Weckhuysen B. M. Catalytic processes monitored at the nanoscale with tip-enhanced Raman spectroscopy. Nat. Nano 7, 583–586 (2012). 10.1038/nnano.2012.131 22902959

[b32] Le Ru E. C. & Etchegoin P. G. Quantifying SERS enhancements. MRS Bull. 38, 631–640 (2013).

[b33] Gooding J. J. & Ciampi S. The molecular level modification of surfaces: from self-assembled monolayers to complex molecular assemblies. Chem. Soc. Rev. 40, 2704–2718 (2011). 21290036 10.1039/c0cs00139b

[b34] Grigorenko A. N., Roberts N. W., Dickinson M. R. & Zhang Y. Nanometric optical tweezers based on nanostructured substrates. Nat. Photonics 2, 365–370 (2008).

[b35] Quidant R. & Girard C. Surface-plasmon-based optical manipulation. Laser Photon. Rev. 2, 47–57 (2008).

[b36] Kim N. H., Meinhart C. D. & Moskovits M. Plasmon-mediated reduction of aqueous platinum ions: the competing roles of field enhancement and hot charge carriers. J. Phys. Chem. C 120, 6750–6755 (2016).

[b37] Minamimoto H. . Visualization of active sites for plasmon-induced electron transfer reactions using photoelectrochemical polymerization of pyrrole. J. Phys. Chem. C 120, 16051–16058 (2016).

[b38] Zhai Y. . Polyvinylpyrrolidone-induced anisotropic growth of gold nanoprisms in plasmon-driven synthesis. Nat. Mater. 15, 889–895 (2016). 27376686 10.1038/nmat4683

[b39] Zheng B. Y. . Distinguishing between plasmon-induced and photoexcited carriers in a device geometry. Nat. Commun. 6, 7797 (2015). 26165521 10.1038/ncomms8797 PMC4510964

[b40] Harutyunyan H. . Anomalous ultrafast dynamics of hot plasmonic electrons in nanostructures with hot spots. Nat. Nano 10, 770–774 (2015). 10.1038/nnano.2015.165 26237345

[b41] Dombi P. . Ultrafast strong-field photoemission from plasmonic nanoparticles. Nano Lett. 13, 674–678 (2013). 23339740 10.1021/nl304365e PMC3573732

[b42] Kale M. J., Avanesian T., Xin H., Yan J. & Christopher P. Controlling catalytic selectivity on metal nanoparticles by direct photoexcitation of adsorbate-metal bonds. Nano Lett. 14, 5405–5412 (2014). 25111312 10.1021/nl502571b

[b43] Zhao L.-B., Chen J.-L., Zhang M., Wu D.-Y. & Tian Z.-Q. Theoretical study on electroreduction of p-nitrothiophenol on silver and gold electrode surfaces. J. Phys. Chem. C 119, 4949–4958 (2015).

[b44] Chalabi H. & Brongersma M. L. Plasmonics: harvest season for hot electrons. Nat. Nano 8, 229–230 (2013). 10.1038/nnano.2013.49 23552114

[b45] Wu K., Chen J., McBride J. R. & Lian T. Efficient hot-electron transfer by a plasmon-induced interfacial charge-transfer transition. Science 349, 632–635 (2015). 26250682 10.1126/science.aac5443

[b46] Nicosia C. & Huskens J. Reactive self-assembled monolayers: from surface functionalization to gradient formation. Mater. Horizons 1, 32–45 (2014).

[b47] Guerra Hernández L. A. . Synergetic light-harvesting and near-field enhancement in multiscale patterned gold substrates. ACS Photonics 2, 1355–1365 (2015).

[b48] Swearer D. F. . Heterometallic antenna-reactor complexes for photocatalysis. Proc. Natl Acad. Sci. USA 113, 8916–8920 (2016). 27444015 10.1073/pnas.1609769113 PMC4987788

[b49] Zhou X. . Selective functionalization of the nanogap of a plasmonic dimer. ACS Photonics 2, 121–129 (2015).

[b50] Galloway C. M. . Plasmon-assisted delivery of single nano-objects in an optical hot spot. Nano Lett. 13, 4299–4304 (2013). 23915079 10.1021/nl402071p

[b51] Jack C. . Spatial control of chemical processes on nanostructures through nano-localized water heating. Nat. Commun. 7, 10946 (2016). 26961708 10.1038/ncomms10946 PMC4792951

[b52] Xie W., Walkenfort B. & Schlücker S. Label-free SERS monitoring of chemical reactions catalyzed by small gold nanoparticles using 3D plasmonic superstructures. J. Am. Chem. Soc. 135, 1657–1660 (2013). 23186150 10.1021/ja309074a

[b53] McPeak K. M. . Plasmonic films can easily be better: rules and recipes. ACS Photonics 2, 326–333 (2015). 25950012 10.1021/ph5004237 PMC4416469

[b54] Ghosh G. Dispersion-equation coefficients for the refractive index and birefringence of calcite and quartz crystals. Opt. Commun. 163, 95–102 (1999).

[b55] Ciddor P. E. Refractive index of air: new equations for the visible and near infrared. Appl. Opt. 35, 1566–1573 (1996). 21085275 10.1364/AO.35.001566

[b56] Kedenburg S., Vieweg M., Gissibl T. & Giessen H. Linear refractive index and absorption measurements of nonlinear optical liquids in the visible and near-infrared spectral region. Opt. Mater. Express 2, 1588–1611 (2012).

[b57] Sundararaman R., Gunceler D., Letchworth-Weaver K., Schwarz K. & Arias T. A. JDFTx. Available from http://jdftx.sourceforge.net (2012). 10.1016/j.softx.2017.10.006 PMC5992620 29892692

