# Supplementary material for: Plasmonic hot electron transport drives nano-localized chemistry
Source: Nat Commun. 2017 Mar 28;8:14880. doi: 10.1038/ncomms14880 (PMC5379059; doi:10.1038/ncomms14880)
Supplement: Supplementary Information — Supplementary Figures, Supplementary Notes and Supplementary References. [file ncomms14880-s1.pdf]

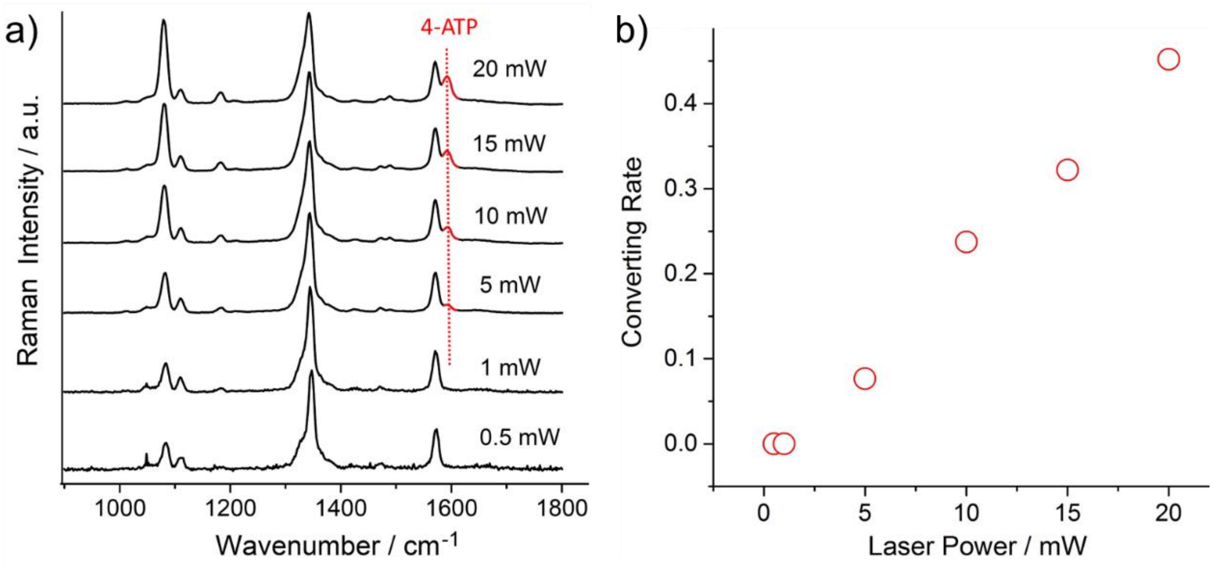

Supplementary Figure 1: Power dependence of hot-electrons reduction of 4-NTP to 4-ATP. a) SERS spectra of the hot-electron reduction reaction using 633 nm laser excitation at different powers and **b)** the corresponding converting rate of 4-NTP to 4-ATP. The experiment was performed on 4-NTP coated AgNPs suspended in a 0.5 M aqueous solution of HCl. The acquisition time of all spectra is 30 s. At low laser powers (0.5 or 1 mW), we can detect SERS signal from 4-NTP but the nitro group on the Ag surface cannot be reduced to amino group. At the maximum laser power of our Raman microscope (20 mW) the converting rate of 4-NTP to 4-ATP is ~45.2% (for the solution of AgNPs). For this range of (relatively low) powers we expect a linear response between laser power and hot-electron production.[1]

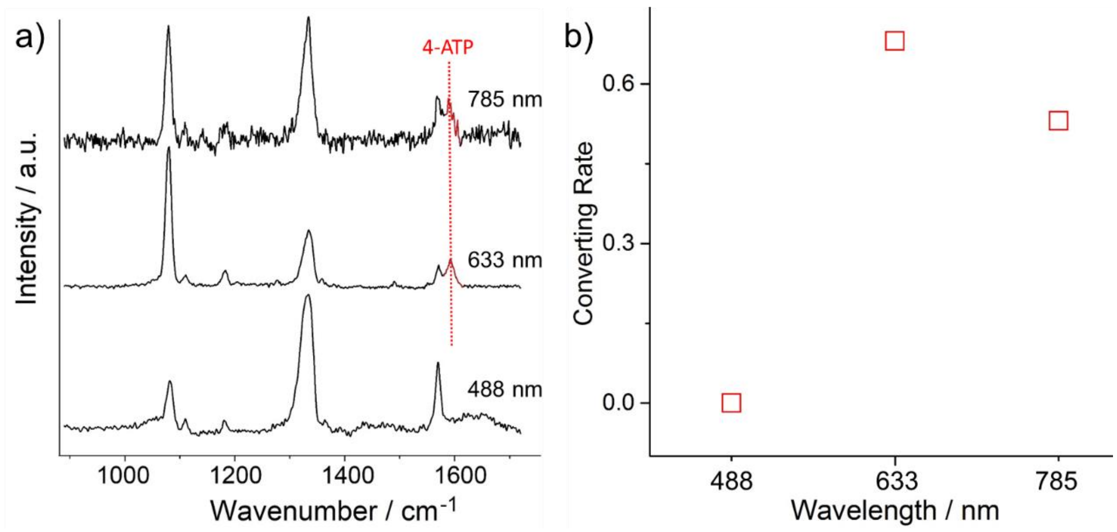

Supplementary Figure 2: Wavelength dependence of hot-electrons reduction of 4-NTP to 4-ATP. a) SERS spectra of the hot-electron reduction reaction using different laser excitation wavelengths. AgNPs (superstructures, LSPR centred at 633 nm) coated with a 4-NTP were suspended in 0.2 M HCl solution for all the experiments. The spectrum obtained at 488 nm was recorded on a Bruker Senterra Raman microscope and a WITec Alpha 300R microscope was used for 633 nm and 785 nm. **b)** Although the experimental conditions are different due to the wavelength dependent-SERS activity of the nanostructures and also the limitation from the setups/software, the 633 nm shows the highest reduction activity with shortest illumination time (10 s for 633 and 785 nm excitation). The SERS bands at 1572 and 1591 cm^{-1} are assigned to the phenol ring vibration modes of 4-NTP and 4-ATP, respectively. The larger relative intensity of these two bands (I_{1591}/I_{1572}) at 633 nm indicates a higher reduction rate from 4-NTP to 4-ATP. In contrast, 488 nm excitation leads to no activity even for longer illumination times (120 s).

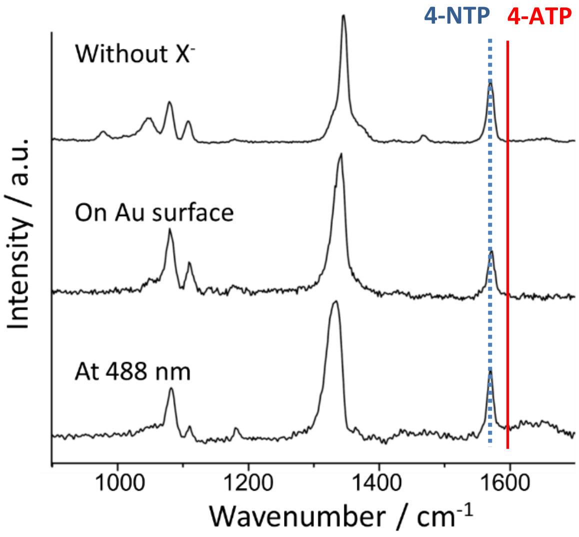

Supplementary Figure 3: Discarding photothermal and photochemical reduction mechanisms. Three negative controls of hot-electron reduction performed in the absence of halide anions (top), on Au surface (middle, without Ag), and at 488 nm excitation (bottom, off resonance), respectively. No reduction (nitro to amino) occurred in these controls. The first and second controls exclude the photothermal mechanism since no reaction was observed when photothermal effects are still present. Additionally, a photochemical effect would have also led to conversion in all the aforementioned controls. Moreover, the absorption peak of 4-NTP on Ag is closer to ~488 nm, thus a photochemical reaction should be favoured for this illumination wavelength.[2] However, we have not detected conversion in these conditions.

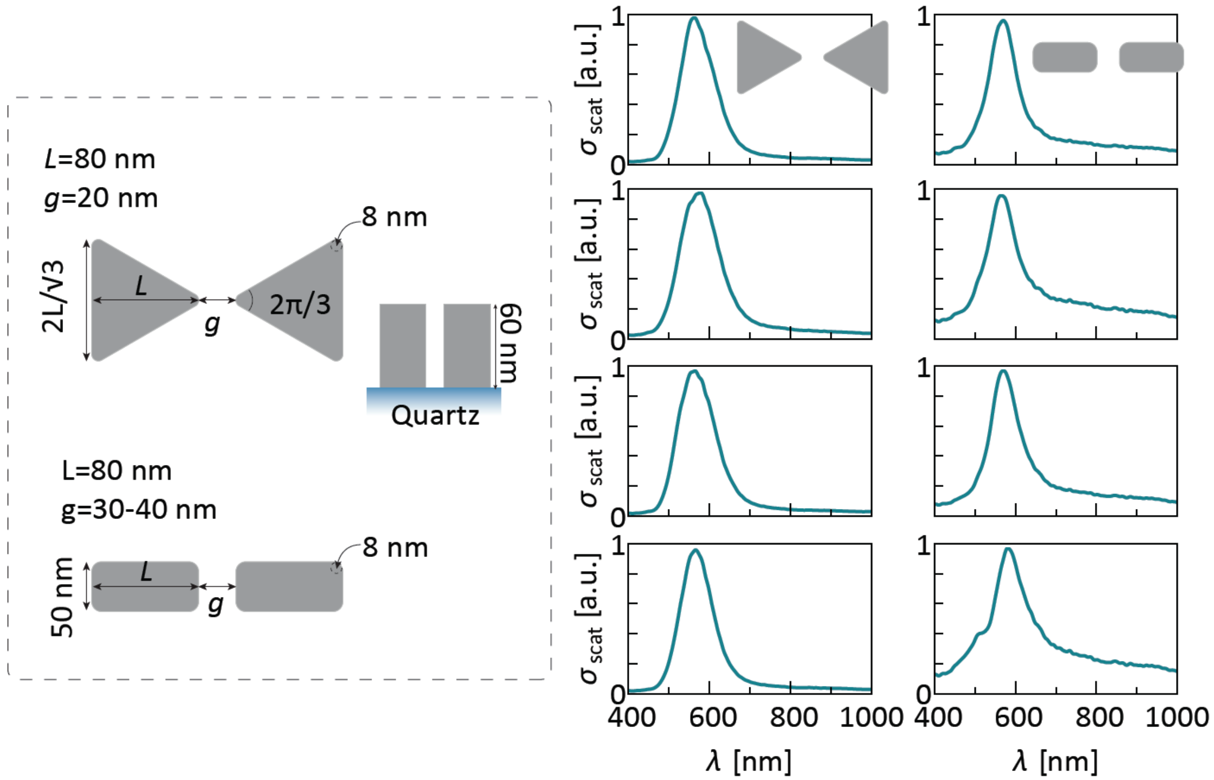

Supplementary Figure 4: Spectral response of the arrays and fabricated dimensions. Schemes of the fabricated Ag nanoantennas. The horizontal length L and gap size g are marked in the figure. The corners of the structures were rounded with an 8 nm radius of curvature to simulate realistic conditions. In both cases, the height of the structures was 60 nm, corresponding to the 60 nm of evaporated Ag. The vertical length was 50 nm for the dimers and $2L/\sqrt{3}$ for the bowties. This length was chosen so as to fix all the internal angles at 2π/3, thus diminishing the amount of free parameters in the simulation. Spectral statistics: The spectra that appear in the main paper are obtained by averaging over many antennae. In this figure four different antenna, both for bowties (left) and dimers (right), are shown. The dark-field spectra were measured in air. We can see that the maximum is around (685 ± 5) nm for both structures, indicating homogeneity in the fabricated structures. SEM images showed that the average gap size for bow-ties was (31±7) nm, whereas for dimers, the average gap size was (27±5) nm.

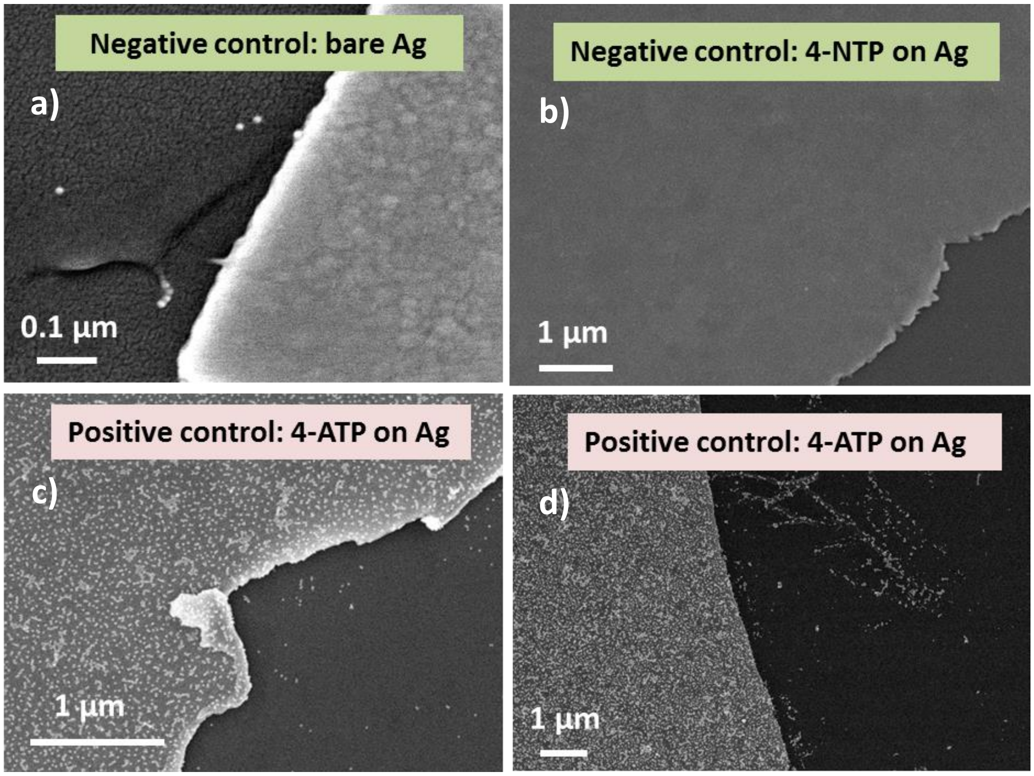

Supplementary Figure 5: Large scale SEM images of negative and positive control experiments. a) SEM image of a bare Ag film (negative control) after reacting with activated AuNPs. No particles can be found on the film or the substrate. **b)** The same result is obtained for 4-NTP coated samples (as mention in the main text). **c-d)** However, full coverage of AuNPs can be obtained over the 4-ATP coated Ag film (positive controls).

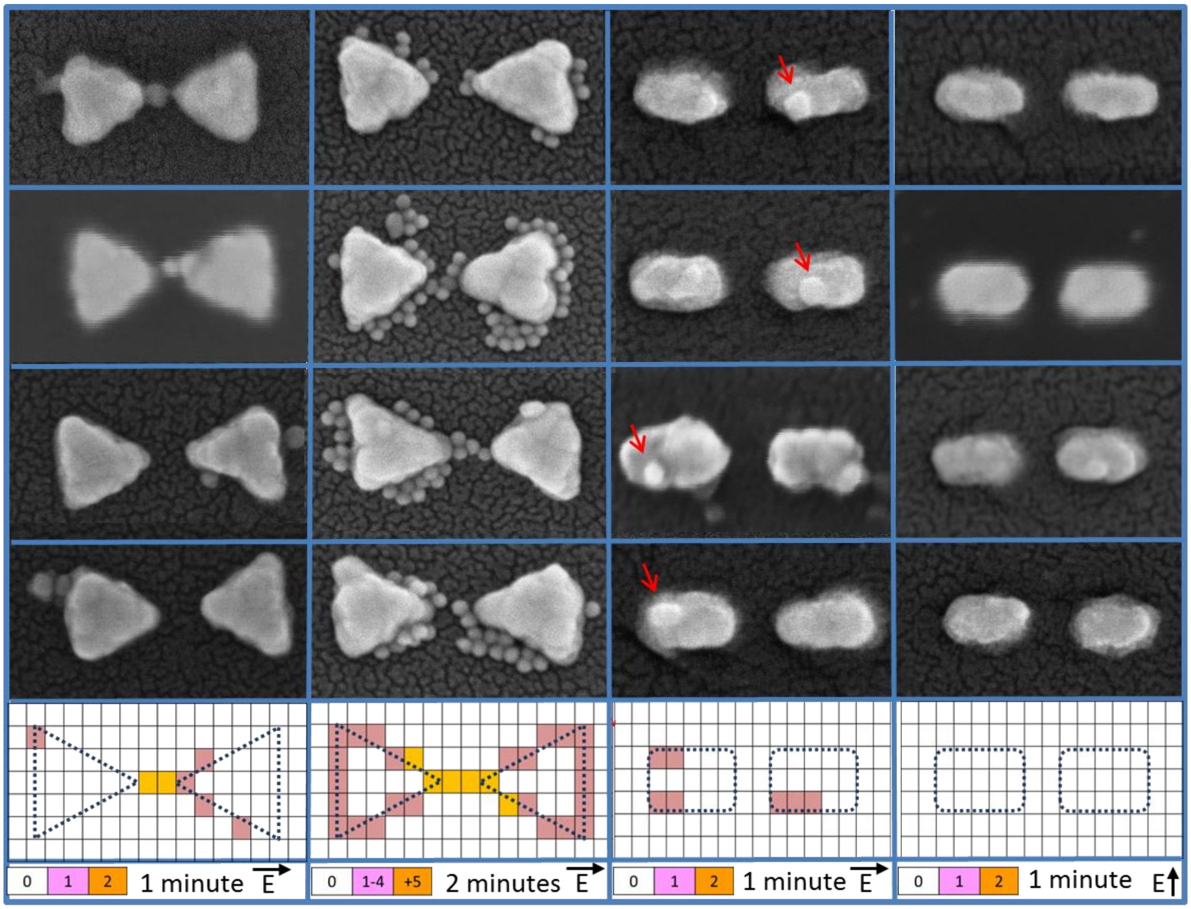

Supplementary Figure 6: Further examples of localized particles. This figure presents additional SEM images of the Ag bow-tie and Ag dimer antennas after hot-electron conversion and coupling to the AuNPs. Statistical analysis shown in the bottom panel of Supplementary Figure 6 (for each condition) was performed only with the SEM images shown in Figure 5 and Supplementary Figure 6 for each case. As it can be seen, this statistic resembles the one presented in Figure 5 (performed over the entire array), but of course in this case the number of localized particles is lower. Only AuNPs in contact with well aligned antennas were considered for the statistics (as described in Supplementary Note 2).

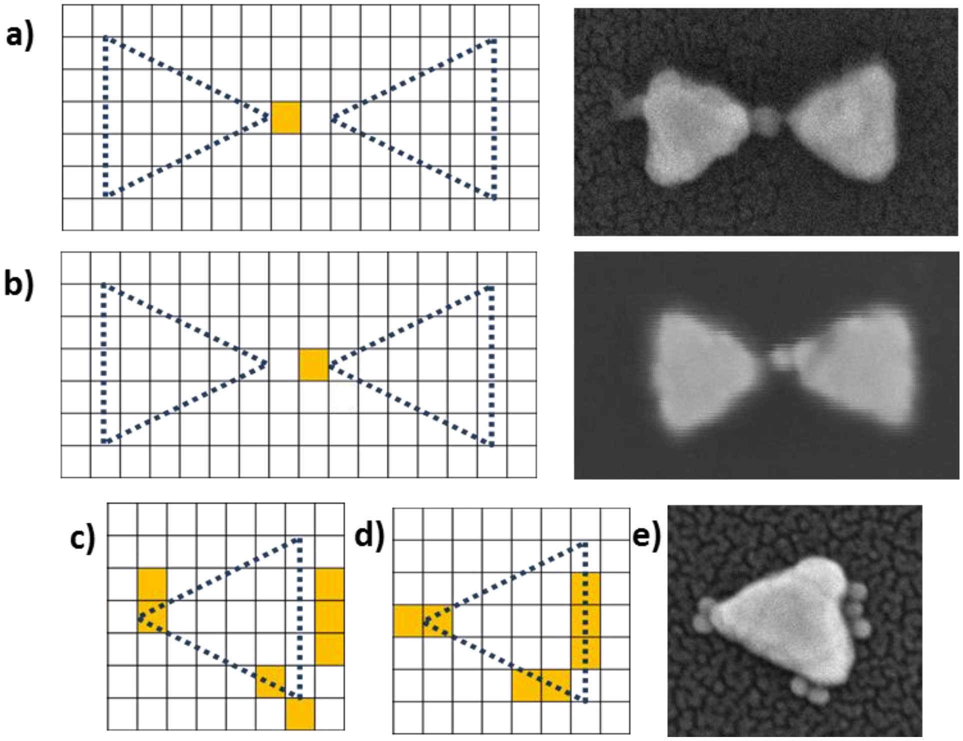

Supplementary Figure 7: Tracking the position and counting the AuNPs. A grid method was used in order to normalize the coordinates of the antennas and obtain the histograms of the localized reporters (AuNPs). **a-b)** Show two simple examples of localizations of one AuNP in each case. **c)** Our attempts to produce scatter plots (without normalizing the antennas' dimensions to the grid) resulted in miss-localization of the AuNPs. This Figure shows the localization (without any normalization) of the AuNPs shown in the SEM image in e). As it can be seen, this does not fairly represent the observed AuNPs (i.e. some AuNPs do no touch the antenna in the grid while this is not the case in the SEM image). Due to imperfections and deviation from ideal sizes (see Supplementary Figure 4), both Ag bow-tie and Ag dimer antennas have to be normalized in order to compute precisely all the localizations in a single grid. To that end, the position of each tip/edge was tracked in SEM as well as the position of the AuNPs. **d)** Normalized localization plot of the SEM image shown in e). **e)** SEM image of one bow-tie's triangle for 2 minutes illumination time at parallel polarization.

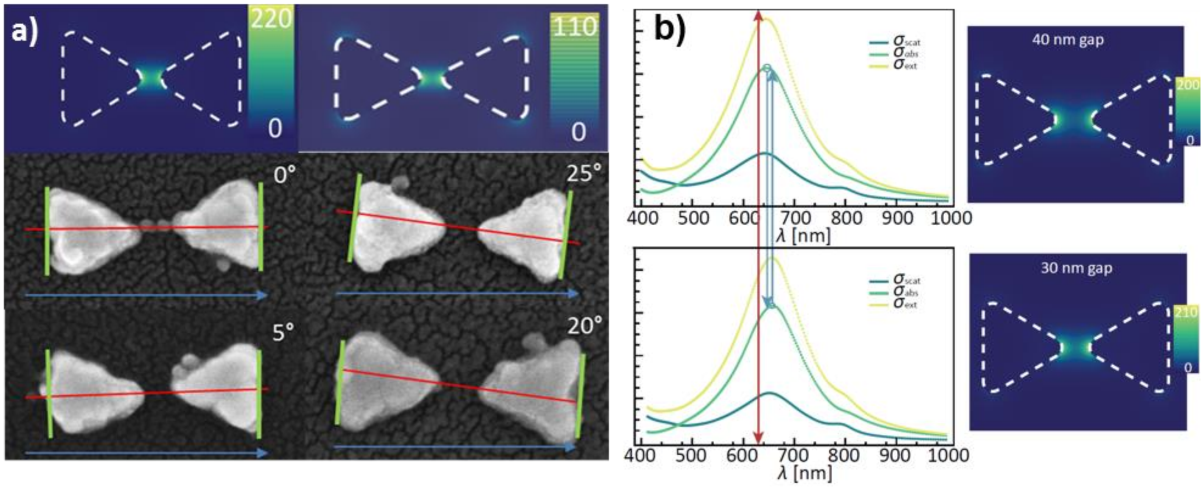

Supplementary Figure 8: Statistics over many antennas. A statistical analysis was performed by a grid-base analysis method. The same analysis was performed over the bar-dimer system and the results are shown in Figure 5c and 5d (bottom panel) of the main text. Further information can be found in Supplementary Notes 2. **a)** Special consideration was made with respect to the main axis orientation as shown in Supplementary Figure 8a, as we have noticed strong dependence on the localization depending on the orientation of the sample respect to the illumination. Important to note is that for parallel polarization, the tips at the hot spot in the Ag bow-ties are the highest EM field regions only (as shown in Figure 2 in the main text), while the tips at the corners and the central one show high EM region for tilted structures. EM field distribution at 45° is shown in at the top in left column. Then, a tilt in the structure would generate a different reactivity pattern as predicted from our calculations and experimental evidence. In order to take into account the orientation and relative position of the antenna, we have also recorded the antenna's position for each case together with the AuNPs. All this information was processed later in order to perform the statistical analysis. **b)** Simulated scattering spectra and near-field distribution for Ag bow-ties antennas with 40 nm (top) and 30 nm (bottom) gaps. The dimensions are kept the same as in Figure 2 of the main text. It can be observed that the field enhancement decreases from 220 for a 30 nm gap to 200 for a 40 nm gap. This difference is in stark contrast to the nearly halved field enhancement in the case of diagonal polarization.

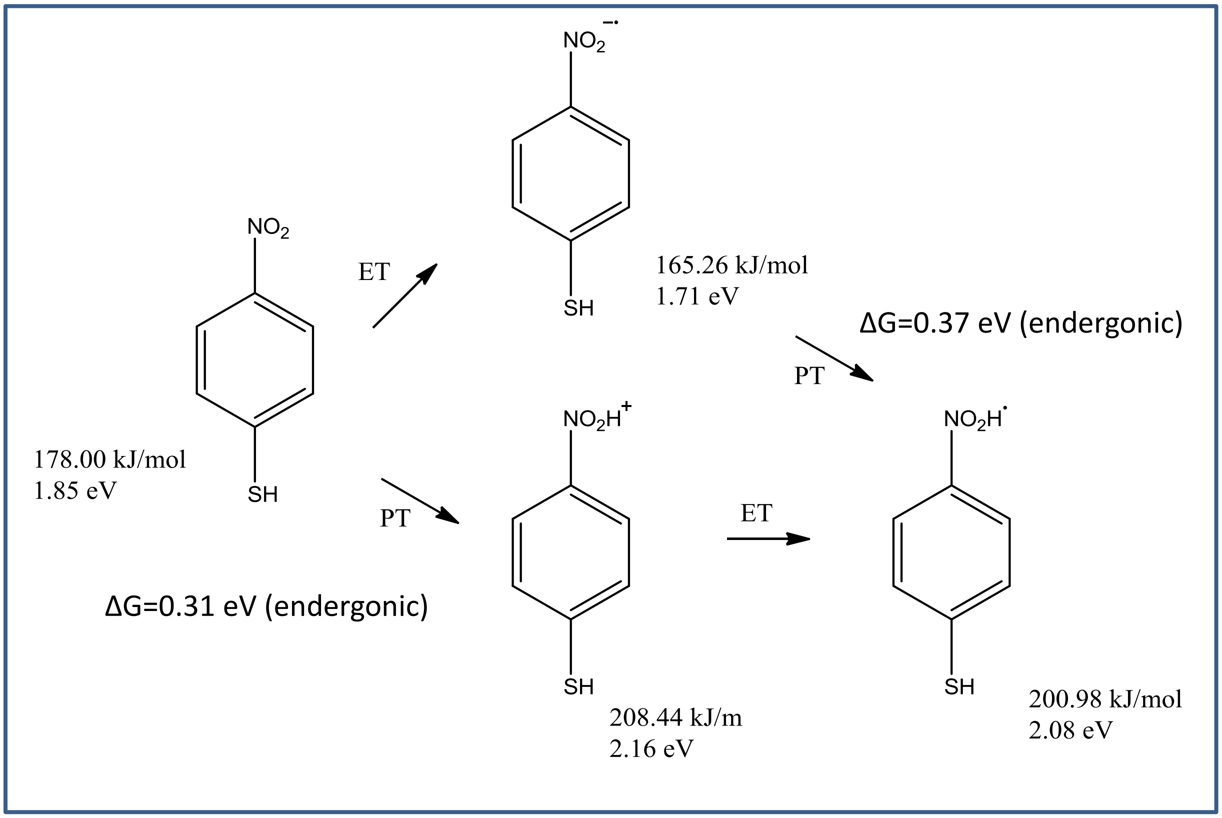

Supplementary Figure 9: Density functional theory (DFT) calculations on possible intermediates. The reaction can either proceeds by a single electron transfer (ET) first followed by a protonation step (PT) or by a protonation step followed by the electron transfer. In both cases the main energetic barrier is associated with the proton transfer (PT) step, with a Gibbs free energy of around 0.35 eV, while values close to 0 eV were found for the electron transfer step (ET). Due to energetic considerations of these possible intermediates the reaction seems to proceed by ET first, without a considerable energy barrier to be overcome followed by a PT step. It is important to note that in order to have a complete molecular picture of the reaction a detail mechanistic study, involving all the intermediates, should be carried out. That complex molecular scenario is out of the scope of the present work.

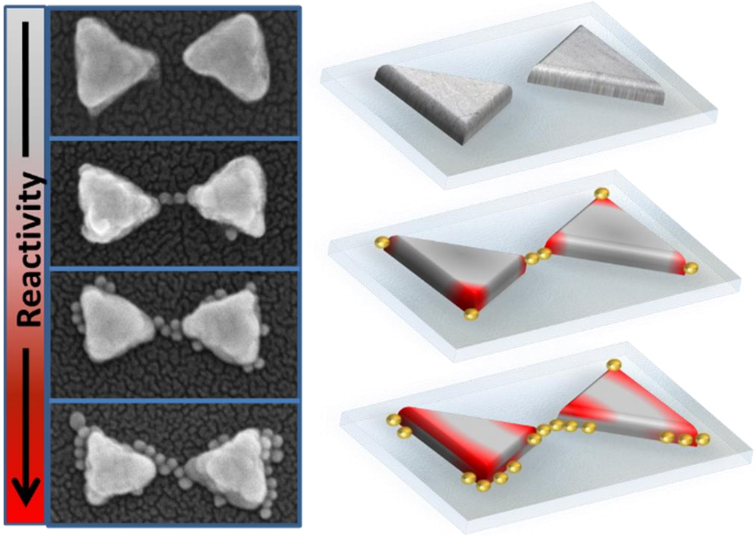

Supplementary Figure 10: Accessing reactive sites in plasmonic antennas. Progressive reactivity in plasmonic nanoantennas due to local hot-electron modification of the surface chemistry. By employing a 4-NTP monolayer that covers the nanoantennas, local amino groups can be generated at the most reactive regions only. A second nanomaterial or molecule can then be guided and self-assembled on the structure. Adjusting the reaction time allows progressive modification.

Supplementary Note 1

Reactions and conditions tested to report on the local hot-electrons conversion. Conjunction of the small AuNPs to the Ag nanoantennas via biotin and streptavidin was tested to report the local hot-electron conversion. To activate the carboxyl group of biotin, EDC and NHS were added to a 10 μM aqueous solution of biotin with a final concentration of 100 and 250 μM, respectively. The converted Ag nanoantenna sample (or 4-ATP coated sample, in positive control experiment) was incubated with the activated biotin solution for 2 hours. After incubation the Ag antennas were washed with water. The obtained biotin conjugated Ag nanoantenna sample was then incubated with streptavidin conjugated 15 nm AuNPs (purchased Nanopartz, with an optical density value of ~1.0) overnight. After all these experimental steps the sample was sputtered with 2-3 nm Pt before SEM imaging. Although the binding efficiency of biotin and streptavidin is very high, there is one additional biotin modification step before the conjugation of streptavidin coated AuNPs and, thus we have a lower coverage of AuNPs on the Ag surface. Therefore we chose the direct EDC/NHS method to report on the local hot-electrons conversion. To reduce the steric hindrance in the EDC/NHS conjugation of MUA coated 15 nm AuNPs, we tested a mix-monolayer of thiol molecules on the Au surface. The AuNPs were first incubated with a mixed solution of 11-MUA and 6-mercapto-1-hexanol, both have the final concentration in the colloid of 5×10^{-9} M. However, this approach shows poor coverage of the AuNPs on Ag surface, probably due to the different binding efficiency of the two thiol molecules to the AuNP surface. Finally, we used the method described in the main text.

Sizes of the AuNPs (reporters). In our approach, the resolution is limited by the size of the AuNPs. We tried AuNPs of different sizes (30 nm, 15 nm and 10 nm) in order to find the threshold for the smallest particles that we could use as probes for detecting the reaction. We faced two problems for the 10 nm AuNPs: increased difficulty in SEM imaging of the probes and aggregation of the particles in solution. As we have described in the main text (see Figure 1), the AuNPs need to be chemically modified (activated) in solution first (by adding EDC/NHS) in order to react later with the amino terminal group of the converted molecules, forming an amide bond. After chemical modification of the AuNPs in solution, two centrifugation steps (purification) were carried out before leaving the AuNPs to react with the converted samples. We noticed that for the 10 nm particles the aggregation in solution (after adding EDC/NHS and centrifugation) was considerably higher than for the 15 nm or 30 nm ones. We modified the reaction conditions (pH, times, with/without agitation, changing the capping from MUA to a carboxylic-acid PEG or mix SAMs), but in all cases the smallest particles were more aggregated than the 15 nm or 30 nm ones. Aggregated particles resulted in big clusters of particles attaching later to the antenna, instead of single probes. For this main reason we did not use smaller particles.

Supplementary Note 2

Statistics over multiple antennas. Statistic (high-resolution SEM imaging) analysis was performed over 100 antennas for each experimental condition (polarization and time of reaction). Each sample (i.e. each experimental conditions tested) is composed of 2 arrays - with 100 antennas each. By employing the

method described in Supplementary Figure 8 we could track the position of the reporter nanoparticles in each antenna and the resulting histograms are presented in Figure 5 (bottom panel) in the main text. For 1 minute reaction and parallel polarization, 30% of the Ag bow-ties antennas have particles attached, while the rest of the antennas do not have particles attached. Shorter illumination times (30 s) resulted in no particles localized.

If the statistic is run without discarding the misaligned antennas, then particles are found mainly at the tips regions. However, we noticed that slightly tilted structures (Supplementary Figure 8b) originate localizations in all the tips, while for better oriented antennas the region near the hot-spot tips are clearly the most reactive ones. Data presented in the main text correspond to this last case (Figure 5, main text). Tilts lower than 20° were considered for the statistic presented in Figure 5 in the main text (in all the histograms). As shown in Supplementary Figures 3 and 8, within the uncertainty in the fabrication in the gap size, we observed that the modification of the simulated field enhancement (i.e. hot-electrons production) was not as strong as that obtained by modifying the angle.

For 2 minutes reaction and parallel polarization, 100% of the bow-ties antennas have particles attached. We have noticed in this case that a small number of particles are not in direct contact with the antennas (i.e. they are attached to another AuNPs). These particles were not considered in the statistics as they were probably aggregated in the solution phase and as such they were assembled as dimers/clusters. Longer illumination times resulted in full conversion.

In the case of bars-dimer, 20% of the antennas have particles on top, while the rest do not have particles attached. For 1 minute reaction and perpendicular polarization, any of the bar-dimer antennas have particles attached. These results were confirmed over different samples and some other reactions were also tested as described before in Supplementary Note 1. These results correspond to the reaction described in the main text (4-NTP to 4-ATP).

Supplementary References

(1) Narang, P.; Sundararaman, R.; Jermyn, A. S.; Goddard, W. A.; Atwater, H. A. Cubic Nonlinearity Driven Up-Conversion in High-Field Plasmonic Hot Carrier Systems. *The Journal of Physical Chemistry C* **2016**, *120*, 21056-21062.

(2) Weitz, D. A.; Garoff, S.; Gersten, J. I.; Nitzan, A. The enhancement of Raman scattering, resonance Raman scattering, and fluorescence from molecules adsorbed on a rough silver surface. *The Journal of Chemical Physics* **1983**, *78*, 5324-5338.
